# Expanding the genetic basis of copy number variation in familial breast cancer

**DOI:** 10.1186/1897-4287-12-15

**Published:** 2014-05-24

**Authors:** Amy L Masson, Bente A Talseth-Palmer, Tiffany-Jane Evans, Desma M Grice, Garry N Hannan, Rodney J Scott

**Affiliations:** 1 Information Based Medicine Program, Hunter Medical Research Institute, University of Newcastle, Newcastle, NSW 2305, Australia; 2 CSIRO Preventative Health Flagship and Animal, CSIRO Food and Health Sciences Division, North Ryde, NSW 2113, Australia; 3 Division of Molecular Medicine, Hunter Area Pathology Service, John Hunter Hospital, Newcastle, NSW 2305, Australia; 4 School of Biomedical Sciences and Pharmacy, Faculty of Health, University of Newcastle, Newcastle, NSW 2308, Australia

**Keywords:** Breast cancer, DNA repair, CNV

## Abstract

**Introduction:**

Familial breast cancer (fBC) is generally associated with an early age of diagnosis and a higher frequency of disease among family members. Over the past two decades a number of genes have been identified that are unequivocally associated with breast cancer (BC) risk but there remain a significant proportion of families that cannot be accounted for by these genes. Copy number variants (CNVs) are a form of genetic variation yet to be fully explored for their contribution to fBC. CNVs exert their effects by either being associated with whole or partial gene deletions or duplications and by interrupting epigenetic patterning thereby contributing to disease development. CNV analysis can also be used to identify new genes and loci which may be associated with disease risk.

**Methods:**

The Affymetrix Cytogenetic Whole Genome 2.7 M (Cyto2.7 M) arrays were used to detect regions of genomic re-arrangement in a cohort of 129 fBC *BRCA1*/*BRCA2* mutation negative patients with a young age of diagnosis (<50 years) compared to 40 unaffected healthy controls (>55 years of age).

**Results:**

CNV analysis revealed the presence of 275 unique rearrangements that were not present in the control population suggestive of their involvement in BC risk. Several CNVs were found that have been previously reported as BC susceptibility genes. This included CNVs in *RPA3*, *NBN* (*NBS1*), *MRE11A* and *CYP19A1* in five unrelated fBC patients suggesting that these genes are involved in BC initiation and/or progression. Of special interest was the identification of *WWOX* and *FHIT* rearrangements in three unrelated fBC patients.

**Conclusions:**

This study has identified a number of CNVs that potentially contribute to BC initiation and/or progression. The identification of CNVs that are associated with known tumour suppressor genes is of special interest that warrants further larger studies to understand their precise role in fBC.

## Introduction

Global cancer statistics identify BC as the most frequently diagnosed cancer (23%) and leading cause of cancer related death (14%) in females [1]. Nearly 27% of these BCs occur in a familial setting typically associated with an earlier age of disease diagnosis and a higher frequency among family members and is termed fBC [2,3]. It is estimated that 5-10% of these families harbor germline mutations or complex genomic changes that render inactive one of four high penetrance genes (*BRCA1*, *BRCA2*, *TP53* or *PTEN*) or moderate penetrance genes (*CHEK2*, *ATM*, *BRIP1* and *PALB2*) [2,4,5]. Associations have also been identified for other genes in fBC including *ATM*, *CASP8*, *CTLA4*, *NBN*, *CYP19A1*, *TERT*, and *XRCC3*[6]. The most recent BC meta-analysis has identified 41 loci and suggests that over 1000 loci may be involved in disease susceptibility [7]. The identification of *BRCA1* and *BRCA2* as susceptibility genes for BC and the more recent addition of *PALB2*, *BRIP1* and *RAD51C*[5] have focused attention on genes associated with double strand break repair (DSBR). There are at least 39 genes implicated in DSBR, all of which could potentially be associated with BC risk. This is analogous to DNA mismatch repair (MMR), where there are at least 21 genes associated with this process, of which four are now routinely assessed and more recently a fifth gene (*POLD1*) has been added to the list [8,9]. Despite the plethora of information regarding genetic loci associated with BC risk, for many fBC cases no genetic predisposition has been identified. Outside the context of gene mutations other mechanisms may be associated with disease development including gene silencing as a result of epigenetic re-programming of BC susceptibility genes (analogous to loss of *EPCAM* and the re-arrangement of the epigenetic profile on chromosome 2, rendering *MSH2* inactive [10,11]), or mutations in genes not yet associated with a predisposition to disease.

One type of genetic alteration that could account for susceptibility is genetic re-arrangements detected as CNVs. CNVs represent a class of structural variation involving regions of duplication or deletion of genomic material that can encompass large stretches of genomic sequence ranging from megabases (Mbs) to a few kilobases (Kb) in size. As a consequence, CNVs can contribute to disease when they incorporate functional gene sequence (coding and promoter regions of genes) or exert more cryptic effects, that could affect epigenetic regulation (methylation, microRNA targets) and non-coding intronic gene sequences [12-23]. Two reports have recently examined CNVs in association with *BRCA1*/*BRCA2* mutation negative fBC patients. The first of these has reported a greater abundance of rare CNVs in fBC patients and suggest that rare CNVs are likely to contain genetic factors associated with BC predisposition, while the second report associated several CNV markers with fBC risk and suggests their use in disease risk assessment [24,25].

The detection of CNVs has historically relied upon the use of DNA arrays, typically comprised of oligonucleotide markers distributed across the whole genome. The resolution of DNA arrays has increased to allow for the detection of genomic rearrangements as small as a few Kb in size. In this study we used the Affymetrix Cyto2.7 M array which provided the highest genomic coverage of any commercially available microarray at the time of assay to assess CNV variation in an fBC cohort. The Cyto2.7 M array contains a combination of 400,000 single nucleotide polymorphisms (SNPs) and >2.1 million copy number probes (average spacing 1395 base pairs (bp)) which together can be used to accurately detect genomic rearrangements.

We conducted a patient-control analysis examining 129 fBC patients and 40 control subjects derived from the same population to identify CNVs which could be associated with the genetic basis of their disease. To date this study represents one of the largest CNV studies of *BRCA1*/*BRCA2* mutation negative fBC patients.

## Materials and methods

### Samples

The study was approved by the University of Newcastle’s Human Research Ethics Committee and the Hunter New England Human Research Ethics Committee. Genomic DNAs were obtained from fBC patients who had given informed consent for their DNA to be used for studies into their disease and control DNA samples from the Hunter Community Study (HCS) [26]. DNA was extracted from whole blood by the salt precipitation method [27].

A cohort of 129 patients clinically diagnosed with early-onset fBC were used in this study. All patients had been diagnosed with BC and were the first individual (proband) of their family to seek genetic testing for mutations in *BRCA1*/*BRCA2*. Mutation screening was performed using Sanger Sequencing and Multiplex ligation-dependant probe amplification (MLPA) analysis. No mutations were identified in any of the patients (*BRCA1*/*BRCA2* mutation negative). The average patient age was calculated to be <40.7 years. Genomic DNA from 40 controls [26] was also utilized in this study. These were healthy (cancer free) individuals aged >55 years at the time of sample collection.

### Genomic array preparation and data processing

The genomic DNA from 129 fBC patients and 40 controls were processed on the Affymetrix Cyto2.7 M array consistent with manufacturer’s protocols. CEL files were analysed in Affymetrix, the Chromosome Analysis Suite (ChAS) (Version CytoB-N1.2.0.232; r4280) using NetAffx Build 30.2 (Hg18) annotation. Quality control (QC) parameters were optimized and validated using a training set of 20 randomly selected samples. All samples were subject to a series of quality cut-off measures: snpQC >1.1 (SNP probe QC based off distances between the distribution of alleles (AA, AB and BB) where larger differences are associated with an increased ability to differentiate genotype; default), mapdQC <0.27 (Median Absolute Pair-wise Difference; CN probe QC based off a reference model; default) and wavinessSd <0.1 (measure of standard deviation in data waviness; the GC content across the genome correlates with average probe intensities i.e. high GC probes are brighter than low GC probes on average, creating waves in the data). CNV regions were assessed according to call confidence, probe count, size and by visual inspection for distinction from normal CN state. Data was also visually inspected to identify regions with low density of markers (Additional file 1: Table S1) which were excluded across all samples. Most thresholds were more stringent than default settings alone in an aim to minimize false-positive CNVs being included in the analysis. CNV regions were filtered across all samples using the following parameters: >90% confidence, autosomes only and a minimum number of 24 probes. Using these parameters the limit of detection was 9.65 Kb across all samples used in the current study. This does not exclude the possibility of CNVs smaller than this from contributing to disease in a proportion of fBC patients.

### CNV and statistical analysis

CNVs in fBC patients and controls were subject to a series of comprehensive analyses which included: (1) interrogation for CNVs residing in or ±100 Kb of 61 genes (associated with DSBR, MMR and BC susceptibility) and 41 SNPs recently reported to be associated with BC risk [6,7,28,29] (see Additional file 1: Tables S2 and S3); (2) comparison of CNVs between fBC patients and controls according to CN occurrence and distribution across the genome; (3) identification of rare CNVs using the Database of Genomic Variants (DGV); and (4) the identification of genes associated with malignancy (non-specific) using the Network of Cancer Genes (Version 3.0) and the Cancer Gene Census (CGC; 15 March 2012) databases [30,31]. Associations (e.g. numbers and sizes of CNVs) were statistically compared using a two tailed un-paired *t*-test Graphpad Prism (Version 6) [32].

### Validation of CNV results

CNV results were validated using pre-designed TaqMan Copy Number (CN) Assays (Applied Biosystems). Up to two CN assays were selected within the CNV region indicated by the Cyto2.7 M array and CN assays, proximal but external to the region were also selected as controls (assay information summarized in Additional file 1: Table S4). A total of 11 samples were run in triplicate comprised of the sample(s) of interest, a calibrator (control) sample with known CN for the region of interest and a no-template-control (NTC). Real-time PCR was conducted according to manufacturer’s protocols using 10 ng of DNA sample in a final reaction volume of 20 μL. The assay was run on the real-time PCR machine (Applied Biosystems 7500; SDS software Version v1.4) according manufacturer’s protocols. The results were exported to CopyCaller v2.0 software (Applied Biosystems) for analysis.

Three CNVs were validated using this secondary independent assay (Additional file 1: Table S5). The CNVs included a CN gain and a CN loss in the *WWOX* gene as well as a CN loss in the *FHIT* gene. Given the high concordance between the CNV calling within the experimental parameters set for this study and the independent copy number assays we considered that it was not necessary to confirm all CNVs using a second independent assay.

## Results

### Array resolution and CNV detection

Analysis of Cyto2.7 M array data revealed a total of 414 CNVs in 169 individuals assessed in this study (Table 1). CNVs detected ranged in size from 9.65 Kb to 1335.06 Kb. There was no difference in the average number of CNVs identified in the patients versus the controls (*p* = 0.75). The average genomic burden of CNVs also did not differ between patients (226.93 Kb) and controls (295.52 Kb), p *= 0.30*; or the average CNV size between patients (76.22 Kb) and controls (106.57 Kb), s, *p = 0.07*.

**Table 1:** Summary of CNV results from the BC patients and control participants

		**CNV Count**			**CNV Size (Kb)**		
		**Total CNVs per group**	**Median CNVs per sample**	**Mean CNVs per sample**	**Total CNV affected genome per group**	**Mean total CNV affected genome per sample**	**Mean size of a CNV**
**Patients**	129	310	2	2.40	29273.63	226.93	76.22
**Controls**	40	104	2	2.60	11820.75	295.52	106.57
***p***	-	-	-	*0.75*	-	*0.30*	*0.07*

### Occurrence and distribution of CNVs in fBC patients

Overall 310 CNVs were identified in fBC patients of which 35 also occurred in controls (Additional file 1: Table S6). Since these regions were represented in the control population they were removed from further analysis. Of the 275 CNVs unique to the patients (Additional file 1: Table S7), 94 have was previously described in the DGV and 39 spanned genomic regions that were common to multiple patients (Table 2). Of these 11 CNVs (located on chromosomes 2, 3, 4, 6, 11, 14, 15, 17 and 18) were common to two patients; three were common to three patients (located on chromosomes 4, 5 and 19); and two were common to four patients (located on chromosomes 3 and 18). Among these, three genomic regions (located chromosomes 6, 11 and 19) were considered novel (not reported in the DGV) and likely to represent regions of potential association with BC risk.

**Table 2:** Genomic regions associated with unique CNVs identified in multiple patients

**Type**	**Chr**	Start (bp)*	End (bp)*	**Size (Kb)**	**Probes**
**2 CNV gains**					
**Gain**	2	13,119,088	13,199,687	80.6	48
**Gain**	2	13,135,013	13,199,687	64.7	43
**Gain**	2	82,055,473	82,163,764	108.3	85
**Gain**	2	82,056,404	82,168,370	112.0	89
**Gain**	3	958,296	1,012,953	54.7	33
**Gain**	3	975,908	1,032,700	56.8	29
**Gain**	6	27,738,385	27,764,062	25.7	26
**Gain**	6	27,742,403	27,770,374	28.0	24
**Gain**	15	79,783,294	79,876,946	93.7	77
**Gain**	15	79,795,446	79,876,343	80.9	70
**Gain**	17	21,503,478	21,648,413	144.9	25
**Gain**	17	21,503,478	21,650,626	147.2	26
**3 CNV gains**					
**Gain**	4	25,672,202	25,703,024	30.8	31
**Gain**	4	25,678,621	25,710,178	31.6	32
**Gain**	4	25,680,434	25,710,412	30.0	31
**Gain**	5	59,749,693	59,807,906	58.2	51
**Gain**	5	59,749,693	59,807,906	58.2	51
**Gain**	5	59,749,693	59,810,944	61.3	52
**Gain**	19	36,911,234	36,939,557	28.3	36
**Gain**	19	36,918,927	36,940,929	22.0	32
**Gain**	19	36,918,927	36,944,555	25.6	36
**2 CNV losses**					
**Loss**	11	95,844,428	95,917,476	73.1	54
**Loss**	11	95,844,428	95,917,476	73.1	54
**Loss**	14	44,229,915	44,294,996	65.1	53
**Loss**	14	44,229,915	44,294,996	65.1	53
**Loss**	17	19,439,549	19,476,055	36.5	28
**Loss**	17	19,439,549	19,476,055	36.5	28
**Loss**	18	1,714,779	1,828,901	114.1	109
**Loss**	18	1,714,779	1,828,901	114.1	109
**4 CNV losses**					
**Loss**	3	166,523,809	166,565,186	41.4	39
**Loss**	3	166,523,809	166,565,186	41.4	39
**Loss**	3	166,523,809	166,566,558	42.8	40
**Loss**	3	166,525,250	166,565,186	39.9	38
**Loss**	18	1,894,368	1,974,284	79.9	63
**Loss**	18	1,894,368	1,974,284	79.9	63
**Loss**	18	1,894,368	1,974,284	79.9	63
**Loss**	18	1,894,368	1,974,284	79.9	63
**2 CNV gain and loss**					
**Gain**	4	160,917,340	161,068,954	151.6	119
**Loss**	4	160,983,513	161,011,918	28.4	29

Probes = number of markers within a CNV segment.

*set at first and last marker associated with the respective CNV.

Of the CNVs unique to patients 160 (58.18%) encompassed genes. A CNV located in *SUPT3H* was also excluded from analysis as it was identified to be affected by a re-arrangement in a control sample and considered unlikely to be associated with disease risk. Therefore a total of 159 genes were associated with a CNV were identified as being unique to the fBC patients and represent genes potentially associated with disease. A total of 24 genes associated with 44 CNVs (gains, losses or both) were identified in multiple individuals (as shown in Table 3): 19 genes, including *LAMB3*, *NBN*, *IL8* and *WWOX*, were affected by a CNV in two individuals; *PIK3R5* and *POU2F3* were affected by a CNV in three individuals; *ARHGEF12* and *TMEM136* were affected by a CNV in four individuals; and *NAMPT* was affected by a CNV in five individuals.

**Table 3:** Genes associated with unique CNVs identified across multiple patients

	**Number of Patients**	**Gene**	**Loci**
**Gains**	2	*B2M*	15q21.1
	2	*DSCAM*	21q22.2
	2	*G0S2*	1q32.2
	2	*GNG2*	14q22.1
	2	*GPR98*	5q14.3
	2	*IL8*	4q13.3
	2	*LAMB3*	1q32.2
	2	*LIMS1*	2q13
	2	*NBN*	8q21.3
	2	*TAGAP*	6q25.3
	2	*TRIM69*	15q21.1
**Both**	2	*CNTN4*	3p26.3
	2	*IMMP2L*	7q31.1
	2	*WWOX*	16q23.1
**Losses**	2	*ACYP2*	2p16.2
	2	*PCDH9*	13q21.32
	2	*SPINT4*	20q13.12
	2	*TSPYL6*	2p16.2
	2	*VAV3*	1p13.3
	3	*PIK3R5*	17p13.1
	3	*POU2F3*	11q23.3
	4	*ARHGEF12*	11q23.3
	4	*TMEM136*	11q23.3
	5	*NAMPT*	7q22.2

### Rare CNVs in fBC patients

There were 95 rare CNVs identified in 42 of the fBC patients. Of these 70 were associated with 78 genes and were found in 27 patients. Out of the 78 genes *SUPT3H* was excluded from further analysis as it was identified in a healthy control subject. Ten genes that were disrupted due to the presence of a CNV had previously been associated with cancer [30,31] including *ARHGAP26*, *ARHGEF12*, *CARD11*, *CPD*, *FAM135B*, *TSHR*, *MLLT11*, *PTK2B*, *RHOH* and *FHIT* (Table 4). The remaining CNVs affecting 67 genes were unique and have not previously been associated with malignancy (listed in Additional file 1: Table S8). These genes potentially represent new candidates that require further investigation.

**Table 4:** Results for the ten CNVs associated with seven patients which affect genes previously associated with cancer

**Genes**	**Dx**	**Type**	**Chr**	**Start (bp)**	**End (bp)**	**Size (Kb)**
***FHIT***	22	Loss	3	60,494,885	60,632,282	137.4
***CARD11***	37	Gain	7	2,946,394	2,996,375	50
***FAM135B***	38	Gain	8	139,259,837	139,306,535	46.7
***ARHGEF12***	51	Gain	11	119,697,081	119,723,342	26.3
***TSHR***	~49	Gain	14	80,659,512	80,669,166	9.7
***MLLT11***	46	Gain	1	149,289,549	149307059	17.5
***CPD***		Gain	17	25,700,671	25,756,973	56.3
***RHOH***	28	Gain	4	39,864,888	39,888,181	23.3
***ARHGAP26***		Gain	5	142,147,309	142,174,652	27.3
***PTK2B***		Gain	8	27,237,115	27,333,842	96.7

Gene, age of patient diagnosis (Dx), CNV type (gain or loss), location (chromosome, start and end) and CNV size are indicated.

### Genomic changes involving BC susceptibility genes or the recently identified BC susceptibility loci

There are at least 61 genes including those involved in DNA DSBR and MMR that could potentially contribute to fBC [6,7,28,29]. CNV data for the 129 fBC patients and 40 controls was screened for genomic re-arrangements within or ±100 Kb either side of these 61 genes. Five patients were identified to harbour CN gains located within or in the vicinity of four genes (Table 5): one within *RPA3* gene; two within the *NBN* gene; one 55.7 Kb upstream of the *MRE11A* gene and one other 89.2 Kb upstream of the *CYP19A1* gene. All gains are predicted to result in disruption of the respective genes’ coding sequence (via the insertion of additional genomic material which is expected to result in loss of function). With respect to the *NBN* gene a CNV loss was also identified in a control residing in a region located 52.6 Kb downstream of the gene but did not appear to be associated with disruption of the coding sequence.

**Table 5:** Search results for regions containing CN gains and CN losses within ±100 Kb the 61 genes associated with BC risk

	**Genes**	**Type**	**Chr**	**Start (bp)**	**End (bp)**	**Size (Kb)**
**Patients**	*RPA3*	Gain	7	7,670,435	7,697,631	27.2
	*NBN*	Gain	8	91,048,149	91,070,004	21.9
	*NBN*	Gain	8	91,050,795	91,088,236	37.4
	55.7 Kb upstream *MRE11A*	Gain	11	93,922,391	93,960,356	38.0
	89.2 Kb upstream *CYP19A1*	Gain	15	49,507,272	49,579,058	71.8
**Control**	52.6 Kb downstream *NBN*	Loss	8	90,913,791	90,962,106	48.3

CNV location (chromosome, start bp and end bp), size (Kb) and type; as well as the gene affected by the variant are indicated.

No CNVs were identified that were located in the same 41 genomic regions that have recently been reported as BC susceptibility loci [7].

**Figure 1:**
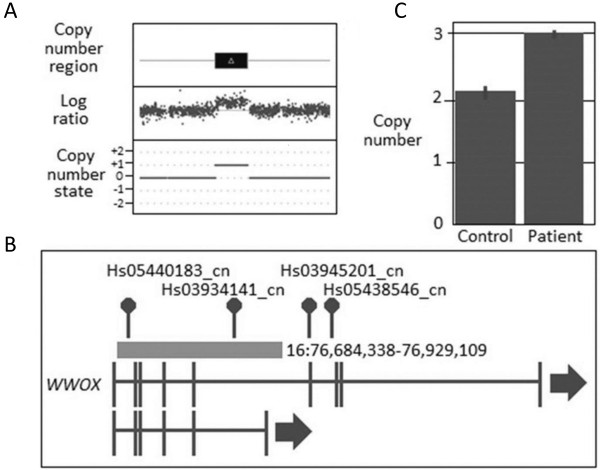
**CNV results for *WWOX *duplication in fBC patient. (A)** CNV profile from Cyto2.7 M array data defining the region of duplication including the genomic state (where 0 = the normal two copies and +1 = one extra copy; **(B)** Location of the duplication within the gene and with respect to the CN assays used in validating the variant; and **(C)** TaqMan CN Validation assay showing the duplication represented by Hs03934141_cn: note the normal two copies of this region identified in the control, confirmation of the aberrant three copies in the fBC patient and the CN range bars associated with the three technical replicates used to validate the CNVs.

**Figure 2:**
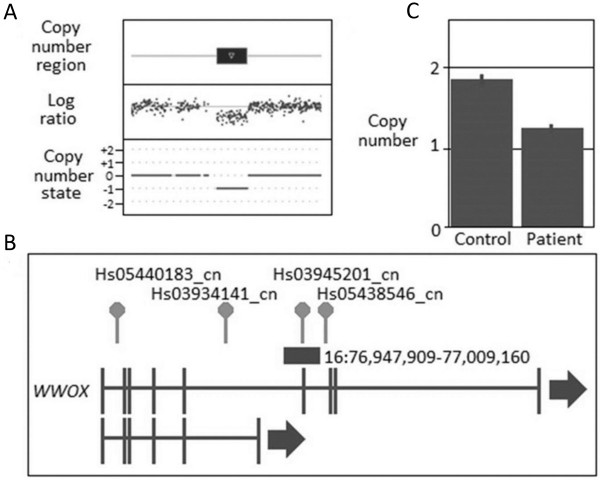
**CNV results for *WWOX *deletion in fBC patient. (A)** CNV profile from Cyto2.7 M array data defining the region of deletion including the genomic state (where 0 = the normal two copies and -1 = one less copy; **(B)** Location of the deletion within the gene and with respect to the CN assays used in validating the variant; and **(C)** TaqMan CN Validation assay showing the deletion represented by Hs03945201_cn: note the normal two copies of this region identified in the control, confirmation of the aberrant one copy in the fBC patient and the CN range bars associated with the three technical replicates used to validate the CNVs.

**Figure 3:**
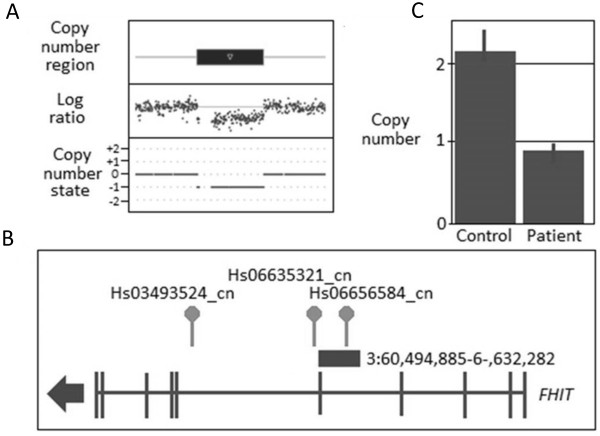
**CNV results for *FHIT *deletion in fBC patient. (A)** CNV profile from Cyto2.7 M array data defining the region of deletion including the genomic state (where 0 = the normal two copies and -1 = one less copy; **(B)** Location of the deletion within the gene and with respect to the CN assays used in validating the variant; and **(C)** TaqMan CN Validation assay showing the deletion represented by Hs06656584_cn: note the normal two copies of this region identified in the control, confirmation of the aberrant one copy in the fBC patient and the CN range bars associated with the three technical replicates used to validate the CNVs.

The identification of a CNV that involved *WWOX* in two unrelated patients (see Table 6, Figures 1 and 2) was of interest as this gene is located in a fragile site (*FRA16D*) associated with cancer development and has been shown to interact with *TP53* and *ACK1*[33] and has recently been reported to be involved in breast carcinogenesis [34,35]. Together, this suggests that loss of function of *WWOX* could potentially be involved in BC susceptibility. One patient harboured a CNV gain that was predicted to disrupt the coding sequence of the gene via the insertion of additional genomic material whereas the other patient had a CNV loss that is expected to result in loss of function. Both of these changes were confirmed using an independent CN assay (see Additional file 1: Table S5). A number of recent reports have also correlated BC development with changes in the *FHIT* gene which similarly to *WWOX* is located in a fragile site (*FRA3B*) and has again been linked to tumour development [36-43]. CNV analysis revealed a CN loss that encompassed *FHIT* (Table 6 and Figure 3) which was confirmed using an independent assay (Additional file 1: Table S5).

**Table 6:** **CNVs associated with fragile site *FRA16D *** **and *FRA3B***

**Chr**	**Start (bp)**	**End (bp)**	**Size (Kb)**	**Gene**	**Probes**	**DGV**
**16**	76,684,338	76,929,109	244.8	*WWOX*	222	Reported
**16**	76,947,909	77,009,160	61.3	*WWOX*	69	Reported
**3**	60,494,885	60,632,282	137.4	*FHIT*	158	

CNV location (chromosome, start bp and end bp) and size (Kb); as well as the confidence score associated with CNV call, the gene affected by the variant, the number of probes used to call the CNV and if the variant has previously been reported in the DGV.

## Discussion

The association between CNVs and fBC is yet to be fully defined. In this study we provide evidence that CNVs are a potential explanation for small but significant number of fBC patients who do not harbour germline mutations in known susceptibility genes.

Genomic resolution provided by microarray technology has increased significantly allowing for the discovery of ever smaller CNVs. The resolution of the array used in this study was limited to the identification of CNVs greater than 9.65 Kb in size, and hence we cannot rule out the potential involvement of smaller CNVs in the aetiology of fBC. There have been a number of technical issues associated with the identification of CNVs that have compounded the difficulties in assessing the role of genomic rearrangements in disease. Different array platforms, software algorithms, batch effects and population stratification influence the accuracy of calls made to and comparisons of CNV data [44-46]. To help in reducing the influence of these effects a set of 40 older population controls was used as the basis to differentiate between CNVs associated with breast cancer and uninformative controls. All samples (both cases and controls) were processed on one platform and analysed using the same analysis software and experimental parameters. Comparison between the number and size of CNVs between patients and controls did not reveal any significant differences between cohorts. It is important to note the limited number of controls utilized in the current study represents a potential bias, however it is reassuring to note that despite this potential limitation, our observations are consistent with two previous reports on fBC (68 patients and 100 controls) and *BRCA1*-associated ovarian cancer (84 patients and 47 controls) [24,47].

We also identified 67 genes associated with novel CNVs that have yet to be linked with BC risk. It is interesting to note that many of these have been implicated in biological processes involving metabolism and biological regulation [48]. This provides the basis for further investigation into expanding the number of genes involved in BC development.

Our study has identified CNVs in close proximity to a number of genes previously associated with BC risk in a fBC cohort: *ARHGEF12* has been proposed to be a candidate tumour suppressor gene in BC whereby its under expression (typically as a result of genomic loss) has been observed in BC cell lines and where re-induction of the gene resulted in reduced cell proliferation and colony formation [49]; Laminin 5 (LN5) genes (including *LAMB3*) have been shown to exhibit reduced expression as a result of epigenetic inactivation in 65% of BC cell lines [50]; *NBN* has been recently reported to be associated with BC risk [6]; and *NAMPT* has been shown to modify the effects of *PARP* inhibitors used in the treatment of triple-negative BCs suggesting the potential for a combination of *NAMPT* and *PARP* inhibitors in the treatment of this disease [51].

Of all the genes affected by a CNV identified in more than one patient, the most frequently reported for BC development has been aberrations in *WWOX*. This tumour suppressor gene has been shown to be critical for normal breast development [34] with mutations in exons 4 to 9 frequently observed in BC tumours [35]. High expression of *WWOX* has been shown to be beneficial in association with tamoxifen treatment [52]. We further evaluated two unrelated fBC patients, one harbouring a CNV gain and the other a CNV loss. In both cases, the genomic rearrangements are predicted to reduce *WWOX* expression and thereby contribute to disease risk. Our results suggest that inherited deficiencies in *WWOX* are associated with disease but we could not demonstrate that these alterations were transmitted across generations due to ethical considerations. Notwithstanding, the frequency at which we have observed variants occurring in this gene (>1.55%) suggests that they may account for a significant proportion of *BRCA1*/*BRCA2* mutation negative fBC patients. Functional studies are required to determine the precise effect of these variants in the alteration of *WWOX* expression and BC development.

The identification of CNVs in close proximity to BC susceptibility genes and loci that either contributes to disease development directly or via more cryptic means expands our understanding of their contribution to disease risk in fBC. Our study identified CNVs residing in three genes *RPA3*, *NBN*, *MRE11A* and *CYP19A1* which supports their involvement in BC [6,28,29,53-56]. Given the predicted disruption of *RPA3*, *NBN*, *MRE11A* and *CYP19A1* it is likely that these variants are associated with disease.

Within our fBC cases we identified several genes within or in close proximity to rare CNVs which have previously been associated with BC: the putative oncogene *MLLT11* (aka *AF1Q*) has been reported to be over expressed in a BC cell line affecting invasive and metastatic potential [57,58]; while *PTK2B* has been shown to be the most frequently lost kinase in sporadic BC tumours and is suggested to contribute to the disease phenotype [59]. Of the rare CNVs associated with malignancy, the gene most frequently associated with BC development is the tumour suppressor *FHIT. FHIT* has been reported multiple times to be genetically and epigenetically modified in breast tumours [36-41]; its expression has been reported to be protective against *HER2*-driven breast tumour development [42]; whereas reduced expression is associated with poor prognosis [43]. A germline intronic deletion in *FHIT* has also been identified in a pancreatic cancer study [60]. Given that we have found a constitutional CNV in *FHIT* we suggest that variants in this gene could also account for a fraction of fBC patients. As we were unable to obtain other family members it remains to be seen if these genomic re-arrangements confer significant disease risk in a family setting rather than being associated with disease progression.

A recent report using 68 patient and 100 controls suggested that rare CNVs may contribute to disease in a small proportion of fBC patients [24]. In contrast to our findings this study reported significantly lower percentages of rare CNVs in fBC patients (4%) compared to the level observed in the current study (30.65%) [24]. The discrepancies in these findings are most likely to be related to differences in sample populations, the type of array used (variation in array coverage and density), as well as the algorithm used by the analysis software [44-46]. These findings reinforce the need to obtain larger cohorts of patients and controls to better understand the contribution of CNVs to breast cancer development.

## Conclusions

This study has revealed that there are a number of CNVs which may contribute to the development of fBC. Several previously reported BC susceptibility genes that include *RPA3*, *NBN*, *MRE11A* and *CYP19A1* were found to be influenced by the presence of a CNV. It was also revealed by this investigation that three unrelated fBC patients harboured CNVs in *WWOX* and *FHIT*. We propose that variants in these genes may account for disease in a significant proportion of fBC patients. Overall the results of this study provide further grounds for further investigation into the presence of CNVs in larger series of fBC patients who do not harbour changes in known breast cancer susceptibility genes.

## Abbreviations

BC: Breast Cancer; bp: Base pair; CGC: Cancer Gene Census; ChAS: Chromosome Analysis Suite (Affymetrix); CN: Copy Number; CNV: Copy Number Variants; Cyto2.7 M: Cytogenetic Whole Genome 2.7 M array; DGV: Database of Genomic Variants; DSB: Double Strand Breat; DSBR: DSB Repair; fBC: Familial Breast Cancer; HCS: Hunter Community Study; Kb: Kilobase; mapd: Median of absolute pair-wise difference; Mb: Megabase; MLPA: Multiplex Ligation-dependant Probe Amplification; MMR: Mismatch Repair; NCG: Network of Cancer Genes; NTC: No template control; QC: Quality control; SNP: Single nucleotide polymorphism; WavinessSd: Waviness standard deviation.

## Competing interests

The authors declare that they have no competing interest.

## Authors’ contributions

ALM conducted the experiments and wrote the first draft of the manuscript. BAT-P, T-JE and DMG provided expertise in data analysis and interpretation as well as revising the manuscript. GNH provided critical review of the manuscript and helped design the experiments. RJS conceived the study, designed the experimental approach and reviewed and approved the final version of the manuscript prior to submission.

## Supplementary Material

Additional file 1: Table S1 Regions of CNV data excluded from CNV analysis due to poor density of probe coverage. **Table S2.** Regions searched for CN gains and CN losses in and in the vicinity of (±100 Kb) of the 61 genes associated with BC risk. **Table S3.** Regions searched for CN gains and CN losses in and in the vicinity of (±100 Kb) of the 41 loci recently reported to be associated with BC risk. **Table S4.** Summary of location and length information for TaqMan copy number assays (Applied Biosystems) used to validate CN duplications and deletions in the *FHIT* and *WWOX* genes. **Table S5.** TaqMan copy number assay results for validation of *WWOX* and *FHIT* CNVs in fBC patients. **Table S6.** List of 35 CNVs identified in patients that are in-common with CNVs identified in controls. **Table S7.** List of 275 CNVs identified in patients that are unique compared to the CNVs identified in controls. **Table S8.** List of 67 genes associated with CNVs uniquely identified in patients and not yet associated with malignancy. Click here for file
