# Supplementary material for: Expanding the genetic basis of copy number variation in familial breast cancer
Source: Hered Cancer Clin Pract. 2014 May 24;12(1):15. doi: 10.1186/1897-4287-12-15 (PMC4064283; doi:10.1186/1897-4287-12-15)
Supplement: Additional file 1: Table S1 — Regions of CNV data excluded from CNV analysis due to poor density of probe coverage. Table S2. Regions searched for CN gains and CN losses in and in the vicinity of (±100 Kb) of the 61 genes associated with BC risk. Table S3. Regions searched for CN gains and CN losses in and in the vicinity of (±100 Kb) of the 41 loci recently reported to be associated with BC risk. Table S4. Summary of location and length information for TaqMan copy number assays (Applied Biosystems) used to validate CN duplications and deletions in the FHIT and WWOX genes. Table S5. TaqMan copy number assay results for validation of WWOX and FHIT CNVs in fBC patients. Table S6. List of 35 CNVs identified in patients that are in-common with CNVs identified in controls. Table S7. List of 275 CNVs identified in patients that are unique compared to the CNVs identified in controls. Table S8. List of 67 genes associated with CNVs uniquely identified in patients and not yet associated with malignancy. [file 1897-4287-12-15-S1.doc]

**Additional file 1**

**Table S1:** Regions of CNV data excluded from CNV analysis due to poor density of probe coverage.

| **Region** | **Centromere** | | **Telomere** | |
| --- | --- | --- | --- | --- |
| **Chr** | p | q | p | q |
| **1** | p11.1, p11.2, p12, p13.1 | q11, q12, q21.1 | p36.33, p36.32, p36.31, p36.23, p36.22, p36.21 | q44 |
| **2** | p11.1, p11.2 | q11.1 | p25.3 | q37.3 |
| **3** | p11.1 | q11.1, q11.2 | none excluded | q29 |
| **4** | p11 | q11 | p16.1, p16.2, p16.3 | q35.2, q35.1 |
| **5** | p12 | q11.1 | p15.33 | q35.3, q35.2, q35.1 |
| **6** | p11.1 | q11.1 | p25.3, p35.2 | q27 |
| **7** | p11.1 | q11.1, q11.21 | p22.3 | q36.3 |
| **8** | p11.1 | q11.1 | p23.3, p23.2, p23.1 | q24.3 |
| **9** | p11.1, p11.2 | q11, q12, q13 | p24.3 | q34.3, q34.2, q34.13, q34.12, q34.11 |
| **10** | p11.1 | q11.1, q11.21, q11.22 | none excluded | q26.3 |
| **11** | p11.11, p11.12 | q11 | p15.5 | q25 |
| **12** | p11.1, p11.21 | q11 | p13.33, p13.32 | q24.33, q24.32, q24.31 |
| **13** | no probes | q11 | no probes | q34 |
| **14** | no probes | q11.1, q11.2 | no probes | q32.33 |
| **15** | no probes | q11.1, q11.2, q12, q13.1, q13.2, q13.3 | no probes | q26.3 |
| **16** | p11.1, p11.2 | q11.1, q11.2 | p13.3 | q24.3, q24.2, q24.1 |
| **17** | none excluded | none excluded | p13.3 | q25.3 |
| **18** | p11.1, p11.21 | q11.1 | none excluded | q23 |
| **19** | p11 | q11 | p13.3 | q13.43 |
| **20** | p11.1, p11.21 | q11.1, q11.21 | none excluded | q13.33 |
| **21** | no probes | q11.1, q11.2 | no probes | q22.3 |
| **22** | no probes | q11.1, q11.21, q11.22 | no probes | q13.33, q13.32 |

**Table S2:** Regions searched for CN gains and CN losses in and in the vicinity of (±100 Kb) of the 61 genes associated with BC risk. Chromosomal position of gene (start and end), gene size and search region (search start and search end) is noted.

| **Gene** | **Chr** | **Start (bp)** | **End (bp)** | **Size (Kb)** | **Search start (bp)** | **Search end (bp)** |
| --- | --- | --- | --- | --- | --- | --- |
| ***RPA2*** | 1 | 28,090,635 | 28,113,823 | 23 | 27,990,635 | 28,213,823 |
| ***PARP1*** | 1 | 224,615,014 | 224,662,424 | 47 | 224,515,014 | 224,762,424 |
| ***EXO1*** | 1 | 240,078,157 | 240,119,671 | 42 | 239,978,157 | 240,219,671 |
| ***MSH2*** | 2 | 47,483,766 | 47,563,864 | 80 | 47,383,766 | 47,663,864 |
| ***MSH6*** | 2 | 47,863,724 | 47,887,596 | 24 | 47,763,724 | 47,987,596 |
| ***PMS1*** | 2 | 190,357,055 | 190,450,600 | 94 | 190,257,055 | 190,550,600 |
| ***CASP8*** | 2 | 201,806,410 | 201,860,679 | 54 | 201,706,410 | 201,960,679 |
| ***CTLA4*** | 2 | 204,440,753 | 204,446,928 | 6 | 204,340,753 | 204,546,928 |
| ***BARD1*** | 2 | 215,301,519 | 215,382,673 | 81 | 215,201,519 | 215,482,673 |
| ***XRCC5*** | 2 | 216,682,264 | 216,779,259 | 97 | 216,582,264 | 216,879,259 |
| ***NHEJ1*** | 2 | 219,648,289 | 219,733,831 | 86 | 219,548,289 | 219,833,831 |
| ***MLH1*** | 3 | 37,009,982 | 37,067,341 | 57 | 36,909,982 | 37,167,341 |
| ***ATRIP*** | 3 | 48,463,221 | 48,482,058 | 19 | 48,363,221 | 48,582,058 |
| ***ATR*** | 3 | 143,650,766 | 143,780,358 | 130 | 143,550,766 | 143,880,358 |
| ***RFC4*** | 3 | 187,990,375 | 188,007,178 | 17 | 187,890,375 | 188,107,178 |
| ***RFC1*** | 4 | 38,965,470 | 39,044,390 | 79 | 38,865,470 | 39,144,390 |
| ***TERT*** | 5 | 1,306,286 | 1,348,162 | 42 | 1,206,286 | 1,448,162 |
| ***MSH3*** | 5 | 79,986,049 | 80,208,390 | 222 | 79,886,049 | 80,308,390 |
| ***XRCC4*** | 5 | 82,409,072 | 82,685,333 | 276 | 82,309,072 | 82,785,333 |
| ***RAD50*** | 5 | 131,920,528 | 132,007,494 | 87 | 131,820,528 | 132,107,494 |
| ***PMS2*** | 7 | 5,979,395 | 6,015,263 | 36 | 5,879,395 | 6,115,263 |
| ***RPA3*** | 7 | 7,643,099 | 7,724,763 | 82 | 7,543,099 | 7,824,763 |
| ***POLD2*** | 7 | 44,120,810 | 44,129,672 | 9 | 44,020,810 | 44,229,672 |
| ***RFC2*** | 7 | 73,283,767 | 73,306,674 | 23 | 73,183,767 | 73,406,674 |
| ***XRCC2*** | 7 | 151,974,519 | 152,004,183 | 30 | 151,874,519 | 152,104,183 |
| ***WRN*** | 8 | 31,010,319 | 31,150,819 | 141 | 30,910,319 | 31,250,819 |
| ***NBN*** | 8 | 91,014,739 | 91,066,075 | 51 | 90,914,739 | 91,166,075 |
| ***ABL1*** | 9 | 132,579,088 | 132,752,883 | 174 | 132,479,088 | 132,852,883 |
| ***PTEN*** | 10 | 89,613,174 | 89,718,512 | 105 | 89,513,174 | 89,818,512 |
| ***DNTT*** | 10 | 98,054,074 | 98,088,311 | 34 | 97,954,074 | 98,188,311 |
| ***POLD4*** | 11 | 66,875,594 | 66,877,593 | 2 | 66,775,594 | 66,977,593 |
| ***POLD3*** | 11 | 73,981,276 | 74,031,413 | 50 | 73,881,276 | 74,131,413 |
| ***MRE11A*** | 11 | 93,790,114 | 93,866,688 | 77 | 93,690,114 | 93,966,688 |
| ***ATM*** | 11 | 107,598,768 | 107,745,036 | 146 | 107,498,768 | 107,845,036 |
| ***ATM*** | 11 | 107,598,768 | 107,745,036 | 146 | 107,498,768 | 107,845,036 |
| ***RFC5*** | 12 | 116,938,890 | 116,954,422 | 16 | 116,838,890 | 117,054,422 |
| ***BRCA2*** | 13 | 31,787,616 | 31,871,809 | 84 | 31,687,616 | 31,971,809 |
| ***RFC3*** | 13 | 33,290,205 | 33,438,695 | 149 | 33,190,205 | 33,538,695 |
| ***LIG4*** | 13 | 107,657,792 | 107,668,717 | 11 | 107,557,792 | 107,768,717 |
| ***PARP2*** | 14 | 19,881,612 | 19,895,903 | 14 | 19,781,612 | 19,995,903 |
| ***MLH3*** | 14 | 74,550,219 | 74,587,988 | 38 | 74,450,219 | 74,687,988 |
| ***XRCC3*** | 14 | 103,233,706 | 103,251,576 | 18 | 103,133,706 | 103,351,576 |
| ***RAD51*** | 15 | 38,774,650 | 38,811,648 | 37 | 38,674,650 | 38,911,648 |
| ***CYP19A1*** | 15 | 49,287,545 | 49,418,087 | 131 | 49,187,545 | 49,518,087 |
| ***BLM*** | 15 | 89,061,582 | 89,159,690 | 98 | 88,961,582 | 89,259,690 |
| ***PALB2*** | 16 | 23,521,983 | 23,560,179 | 38 | 23,421,983 | 23,660,179 |
| ***RPA1*** | 17 | 1,680,022 | 1,749,598 | 70 | 1,580,022 | 1,849,598 |
| ***TP53*** | 17 | 7,512,444 | 7,531,588 | 19 | 7,412,444 | 7,631,588 |
| ***LIG3*** | 17 | 30,331,650 | 30,356,201 | 25 | 30,231,650 | 30,456,201 |
| ***BRCA1*** | 17 | 38,449,839 | 38,531,026 | 81 | 38,349,839 | 38,631,026 |
| ***BRIP1*** | 17 | 57,114,766 | 57,295,537 | 180 | 57,014,766 | 57,395,537 |
| ***RBBP8*** | 18 | 18,767,292 | 18,860,447 | 93 | 18,667,292 | 18,960,447 |
| ***STK11*** | 19 | 1,156,797 | 1,179,434 | 23 | 1,056,797 | 1,279,434 |
| ***XRCC1*** | 19 | 48,739,303 | 48,771,570 | 32 | 48,639,303 | 48,871,570 |
| ***LIG1*** | 19 | 53,310,514 | 53,365,372 | 55 | 53,210,514 | 53,465,372 |
| ***PNKP*** | 19 | 55,056,272 | 55,062,630 | 6 | 54,956,272 | 55,162,630 |
| ***POLD1*** | 19 | 55,579,404 | 55,613,083 | 34 | 55,479,404 | 55,713,083 |
| ***PCNA*** | 20 | 5,043,598 | 5,055,268 | 12 | 4,943,598 | 5,155,268 |
| ***BCR*** | 22 | 21,852,551 | 21,990,224 | 138 | 21,752,551 | 22,090,224 |
| ***CHEK2*** | 22 | 27,413,730 | 27,467,822 | 54 | 27,313,730 | 27,567,822 |
| ***XRCC6*** | 22 | 40,347,240 | 40,389,998 | 43 | 40,247,240 | 40,489,998 |

**Table S3:** Regions searched for CN gains and CN losses in and in the vicinity of (±100 Kb) of the 41 loci recently reported to be associated with BC risk. SNP, position of loci (Chromosome and location) and affected genes are noted.

| **SNP** | **Chr** | **Position** | **Genes** | **Search Start (bp)** | **Search End (bp)** |
| --- | --- | --- | --- | --- | --- |
| **rs616488** | 1 | 10,488,802 | *PEX14* | 10,388,802 | 10,588,802 |
| **rs11552449** | 1 | 114,249,912 | *PTPN22-BCL2L15-AP4B1-DCLRE1B-HIPK1* | 114,149,912 | 114,349,912 |
| **rs4849887** | 2 | 120,961,592 | *None* | 120,861,592 | 121,061,592 |
| **rs2016394** | 2 | 172,681,217 | *METAP1D-DLX1-DLX2* | 172,581,217 | 172,781,217 |
| **rs1550623** | 2 | 173,921,140 | *CDCA7* | 173,821,140 | 174,021,140 |
| **rs16857609** | 2 | 218,004,753 | *DIRC3* | 217,904,753 | 218,104,753 |
| **rs6762644** | 3 | 4,717,276 | *ITPR1-EGOT* | 4,617,276 | 4,817,276 |
| **rs12493607** | 3 | 30,657,943 | *TGFBR2* | 30,557,943 | 30,757,943 |
| **rs9790517** | 4 | 106,304,227 | *TET2* | 106,204,227 | 106,404,227 |
| **rs6828523** | 4 | 176,083,001 | *ADAM29* | 175,983,001 | 176,183,001 |
| **rs10472076** | 5 | 58,219,818 | *RAB3C* | 58,119,818 | 58,319,818 |
| **rs1353747** | 5 | 58,373,238 | *PDE4D* | 58,273,238 | 58,473,238 |
| **rs1432679** | 5 | 158,176,661 | *EBF1* | 158,076,661 | 158,276,661 |
| **rs11242675** | 6 | 1,263,878 | *FOXQ1* | 1,163,878 | 1,363,878 |
| **rs204247** | 6 | 13,830,502 | *RANBP9* | 13,730,502 | 13,930,502 |
| **rs720475** | 7 | 143,705,862 | *ARHGEF5-NOBOX* | 143,605,862 | 143,805,862 |
| **rs9693444** | 8 | 29,565,535 | *None* | 29,465,535 | 29,665,535 |
| **rs6472903** | 8 | 76,392,856 | *None* | 76,292,856 | 76,492,856 |
| **rs2943559** | 8 | 76,580,492 | *HNF4G* | 76,480,492 | 76,680,492 |
| **rs11780156** | 8 | 129,263,823 | *MIR1208* | 129,163,823 | 129,363,823 |
| **rs10759243** | 9 | 109,345,936 | *None* | 109,245,936 | 109,445,936 |
| **rs7072776** | 10 | 22,072,948 | *MLLT10-DNAJC1* | 21,972,948 | 22,172,948 |
| **rs11814448** | 10 | 22,355,849 | *DNAJC1* | 22,255,849 | 22,455,849 |
| **rs7904519** | 10 | 114,763,917 | *TCF7L2* | 114,663,917 | 114,863,917 |
| **rs11199914** | 10 | 123,083,891 | *None* | 122,983,891 | 123,183,891 |
| **rs3903072** | 11 | 65,339,642 | *DKFZp761E198-OVOL1-SNX32-CFL1-MUS81* | 65,239,642 | 65,439,642 |
| **rs11820646** | 11 | 128,966,381 | *None* | 128,866,381 | 129,066,381 |
| **rs12422552** | 12 | 14,305,198 | *None* | 14,205,198 | 14,405,198 |
| **rs17356907** | 12 | 94,551,890 | *NTN4* | 94,451,890 | 94,651,890 |
| **rs11571833** | 13 | 31,870,626 | *BRCA2-N4BP2L1-N4BP2L2* | 31,770,626 | 31,970,626 |
| **rs2236007** | 14 | 36,202,520 | *PAX9-SLC25A21* | 36,102,520 | 36,302,520 |
| **rs2588809** | 14 | 67,730,181 | *RAD51L1* | 67,630,181 | 67,830,181 |
| **rs941764** | 14 | 90,910,822 | *CCDC88C* | 90,810,822 | 91,010,822 |
| **rs17817449** | 16 | 52,370,868 | *MIR1972-2-FTO* | 52,270,868 | 52,470,868 |
| **rs13329835** | 16 | 79,208,306 | *CDYL2* | 79,108,306 | 79,308,306 |
| **rs527616** | 18 | 22,591,422 | *None* | 22,491,422 | 22,691,422 |
| **rs1436904** | 18 | 22,824,665 | *CHST9* | 22,724,665 | 22,924,665 |
| **rs4808801** | 19 | 18,432,141 | *SSBP4-ISYNA1-ELL* | 18,332,141 | 18,532,141 |
| **rs3760982** | 19 | 48,978,353 | *C19orf61-KCNN4-LYPD5-ZNF283* | 48,878,353 | 49,078,353 |
| **rs132390** | 22 | 27,951,477 | *EMID1-RHBDD3-EWSR1* | 27,851,477 | 28,051,477 |
| **rs6001930** | 22 | 39,206,180 | *MKL1* | 39,106,180 | 39,306,180 |

**Table S4: Summary of location and length information for TaqMan copy number assays (Applied Biosystems) used to validate CN duplications and deletions in the *FHIT* and *WWOX* genes.**

| **Gene** | **CN Assay** | **Location** | **Length (bp)** |
| --- | --- | --- | --- |
| ***FHIT*** | Hs06656584_cn | 3:60,598,999 | 99 |
|  | Hs06635321_cn | 3:60,518,932 | 110 |
|  | Hs03493524_cn | 3:60,001,448 | 102 |
| ***WWOX*** | Hs03934141_cn | 16:78,303,363 | 109 |
|  | Hs03945201_cn | 16:78,401,608 | 92 |
|  | Hs05440183_cn | 16:78,201,830 | 102 |
|  | Hs05438546_cn | 16:78,428,046 | 110 |

**Table S5:** TaqMan copy number assay results for validation of *WWOX* and *FHIT* CNVs in fBC patients. Note with respect to each of the three assays, the calculated CN and predicted CN.

| **Assay** | **Sample** | **Calc CN** | **Pred CN** |
| --- | --- | --- | --- |
| **Hs05540183_cn** | Control1 | 2.06 | 2 |
|  | 5 | 1.99 | 2 |
|  | fBC2 | 1.93 | 2 |
|  | fBC3 | 2.79 | 3 |
|  | 6 | 2.09 | 2 |
|  | 7 | 2.11 | 2 |
|  | 8 | 2.01 | 2 |
|  | 9 | 1.87 | 2 |
|  | Control2 | 2.08 | 2 |
|  | 12 | 1.8 | 2 |
|  | 13 | 1.95 | 2 |
| **Hs03931414_cn** | Control1 | 2.09 | 2 |
|  | 5 | 2.13 | 2 |
|  | fBC2 | 2 | 2 |
|  | fBC3 | 3 | 3 |
|  | 7 | 2.07 | 2 |
|  | 8 | 2.09 | 2 |
|  | 10 | 1.75 | 2 |
|  | 14 | 1.66 | 2 |
|  | Control2 | 2.18 | 2 |
|  | 12 | 1.73 | 2 |
|  | 13 | 1.84 | 2 |
| **Hs05438546_cn** | Control1 | 2.12 | 2 |
|  | 5 | 2.19 | 2 |
|  | fBC2 | 1.85 | 2 |
|  | fBC3 | 1.81 | 2 |
|  | 6 | 1.96 | 2 |
|  | 7 | 2.13 | 2 |
|  | 8 | 2.02 | 2 |
|  | 10 | 1.89 | 2 |
|  | Control2 | 2.13 | 2 |
|  | 12 | 1.65 | 2 |
|  | 13 | 1.96 | 2 |
| **Hs03945201_cn** | Control1 | 1.84 | 2 |
|  | 5 | 1.83 | 2 |
|  | fBC2 | 1.25 | 1 |
|  | fBC3 | 2.48 | 3 |
|  | 7 | 1.81 | 2 |
|  | 8 | 1.85 | 2 |
|  | 10 | 2.19 | 2 |
|  | 14 | 2.18 | 2 |
|  | Control2 | 1.87 | 2 |
|  | 12 | 2.1 | 2 |
|  | 13 | 1.83 | 2 |
| **Hs03493524_cn** | Control1 | 2.08 | 2 |
|  | 5 | 2.33 | 2 |
|  | fBC1 | 2.15 | 2 |
|  | 6 | 2.05 | 2 |
|  | 7 | 1.98 | 2 |
|  | 8 | 1.98 | 2 |
|  | 9 | 1.47 | 2 |
|  | 10 | 1.93 | 2 |
|  | Control2 | 2.15 | 2 |
|  | 12 | 1.84 | 2 |
|  | 13 | 1.91 | 2 |
| **Hs06635321_cn** | Control1 | 2.01 | 2 |
|  | 5 | 2.05 | 2 |
|  | fBC1 | 1.87 | 2 |
|  | 6 | 1.91 | 2 |
|  | 7 | 2.18 | 2 |
|  | 8 | 2.17 | 2 |
|  | 9 | 1.63 | 2 |
|  | 10 | 1.84 | 2 |
|  | Control2 | 2.21 | 2 |
|  | 12 | 1.79 | 2 |
|  | 13 | 1.94 | 2 |
| **Hs06656584_cn** | Control1 | 2.14 | 2 |
|  | 5 | 2.25 | 2 |
|  | fBC1 | 0.9 | 1 |
|  | 6 | 1.91 | 2 |
|  | 7 | 2.17 | 2 |
|  | 8 | 2.12 | 2 |
|  | 9 | 1.45 | 2 |
|  | 10 | 1.91 | 2 |
|  | Control2 | 2.13 | 2 |
|  | 12 | 1.83 | 2 |
|  | 13 | 1.94 | 2 |

**Table S6:** List of 35 CNVs identified in patients that are in-common with CNVs identified in controls. Chromosomal position (start bp and end bp), CNV size (Kb), CN type, gene(s) affected, number of probes detecting the region and if the region has been reported in the DGV is noted for all CNVs.

| **Chr** | **Start (bp)** | **End (bp)** | **Size (Kb)** | **Genes** | **Probes** | **DGV** |
| --- | --- | --- | --- | --- | --- | --- |
| **2** | 57,250,791 | 57,293,667 | 42.876 |  | 32 | Reported |
| **2** | 57,252,457 | 57,293,667 | 41.21 |  | 30 | Reported |
| **2** | 74,764,420 | 74,833,462 | 69.042 |  | 49 | Reported |
| **3** | 4,094,787 | 4,206,861 | 112.074 |  | 89 | Reported |
| **3** | 4,204,322 | 4,253,862 | 49.54 |  | 69 | Reported |
| **3** | 174,722,146 | 174,772,293 | 50.147 | *NLGN1* | 49 | Reported |
| **4** | 162,172,629 | 162,223,002 | 50.373 |  | 44 | Reported |
| **5** | 32,144,416 | 32,204,638 | 60.222 | *PDZD2, GOLPH3* | 55 | Reported |
| **5** | 32,146,572 | 32,202,727 | 56.155 | *PDZD2, GOLPH3* | 51 | Reported |
| **5** | 104,461,414 | 104,529,373 | 67.959 | *RAB9P1* | 40 | Reported |
| **5** | 104,461,414 | 104,529,373 | 67.959 | *RAB9P1* | 40 | Reported |
| **5** | 104,461,414 | 104,529,373 | 67.959 | *RAB9P1* | 40 | Reported |
| **6** | 45,216,636 | 45,240,255 | 23.619 | *SUPT3H* | 31 |  |
| **6** | 124,472,813 | 124,512,504 | 39.691 | *NKAIN2* | 55 | Reported |
| **6** | 124,474,038 | 124,512,504 | 38.466 | *NKAIN2* | 52 | Reported |
| **6** | 124,475,557 | 124,512,504 | 36.947 | *NKAIN2* | 51 | Reported |
| **7** | 8,794,054 | 8,829,923 | 35.869 |  | 34 | Reported |
| **7** | 8,794,054 | 8,831,295 | 37.241 |  | 35 | Reported |
| **7** | 8,794,054 | 8,831,295 | 37.241 |  | 35 | Reported |
| **7** | 8,794,054 | 8,831,295 | 37.241 |  | 35 | Reported |
| **7** | 142,537,643 | 142,600,511 | 62.868 | *PIP, TAS2R39* | 45 | Reported |
| **7** | 142,537,643 | 142,600,511 | 62.868 | *PIP, TAS2R39* | 45 | Reported |
| **7** | 142,537,643 | 142,600,511 | 62.868 | *PIP, TAS2R39* | 45 | Reported |
| **7** | 142,537,643 | 142,605,852 | 68.209 | *PIP, TAS2R39* | 46 | Reported |
| **8** | 137,748,050 | 137,919,630 | 171.58 |  | 57 | Reported |
| **8** | 137,751,665 | 137,937,181 | 185.516 |  | 61 | Reported |
| **9** | 28,620,385 | 28,695,405 | 75.02 | *LINGO2* | 96 | Reported |
| **12** | 7,883,487 | 8,015,788 | 132.301 | *SLC2A14, SLC2A3* | 34 | Reported |
| **12** | 7,895,002 | 8,015,788 | 120.786 | *SLC2A14, SLC2A3* | 31 | Reported |
| **15** | 85,626,838 | 85,671,184 | 44.346 |  | 48 | Reported |
| **15** | 85,626,838 | 85,671,184 | 44.346 |  | 48 | Reported |
| **15** | 85,626,838 | 85,671,184 | 44.346 |  | 48 | Reported |
| **20** | 14,729,522 | 14,774,064 | 44.542 | *MACROD2* | 57 | Reported |
| **20** | 40,612,302 | 40,675,302 | 63 | *PTPRT* | 98 | Reported |
| **21** | 21,268,131 | 21,367,798 | 99.667 | *NCAM2* | 114 | Reported |

**Table S7:** List of 275 CNVs identified in patients that are unique compared to the CNVs identified in controls. Chromosomal position (start bp and end bp), CNV size (Kb), CN type, gene(s) affected, number of probes detecting the region and if the region has been reported in the DGV is noted for all CNVs.

| **Chr** | **Start (bp)** | **End (bp)** | **Size (Kb)** | **Genes** | **Probes** | **DGV** |
| --- | --- | --- | --- | --- | --- | --- |
| **1** | 23,565,643 | 23,606,118 | 40.475 | *ZNF436, C1orf213, TCEA3* | 40 |  |
| **1** | 28,308,941 | 28,347,358 | 38.417 |  | 29 |  |
| **1** | 40,622,494 | 40,649,739 | 27.245 | *SMAP2* | 38 |  |
| **1** | 70,873,428 | 70,903,760 | 30.332 |  | 31 | Reported |
| **1** | 97,167,318 | 97,192,309 | 24.991 |  | 25 |  |
| **1** | 105,826,320 | 105,890,887 | 64.567 |  | 59 | Reported |
| **1** | 107,981,275 | 108,054,335 | 73.06 | *VAV3* | 87 | Reported |
| **1** | 107,981,275 | 108,054,335 | 73.06 | *VAV3* | 87 | Reported |
| **1** | 149,289,549 | 149,307,059 | 17.51 | *C1orf56, CDC42SE1, MLLT11* | 25 |  |
| **1** | 159,585,380 | 159,612,968 | 27.588 | *SDHC, LOC642502, C1orf192* | 44 | Reported |
| **1** | 164,812,553 | 164,882,918 | 70.365 | *FMO9P* | 100 | Reported |
| **1** | 187,592,702 | 187,810,312 | 217.61 |  | 156 | Reported |
| **1** | 189,132,919 | 189,209,948 | 77.029 |  | 79 | Reported |
| **1** | 194,093,254 | 194,141,479 | 48.225 |  | 49 | Reported |
| **1** | 207,852,879 | 207,924,028 | 71.149 | *CAMK1G, LAMB3, G0S2* | 32 |  |
| **1** | 207,890,364 | 207,939,169 | 48.805 | *LAMB3, G0S2, HSD11B1* | 34 |  |
| **1** | 215,384,816 | 215,436,512 | 51.696 |  | 70 | Reported |
| **1** | 236,718,583 | 236,845,495 | 126.912 |  | 106 | Reported |
| **2** | 9,934,431 | 10,266,015 | 331.584 | *TAF1B, GRHL1, KLF11, CYS1, RRM2, C2orf48* | 91 | Reported |
| **2** | 13,119,088 | 13,199,687 | 80.599 |  | 48 | Reported |
| **2** | 13,135,013 | 13,199,687 | 64.674 |  | 43 | Reported |
| **2** | 32,482,418 | 33,185,667 | 703.249 | *BIRC6, TTC27, LOC285045, LOC100271832, LTBP1* | 482 | Reported |
| **2** | 38,671,724 | 38,801,802 | 130.078 | *HNRPLL, GALM* | 54 | Reported |
| **2** | 42,168,199 | 42,224,991 | 56.792 |  | 37 | Reported |
| **2** | 54,185,498 | 54,377,579 | 192.081 | *ACYP2, TSPYL6* | 173 |  |
| **2** | 54,185,498 | 54,377,579 | 192.081 | *ACYP2, TSPYL6* | 173 |  |
| **2** | 69,979,266 | 70,214,967 | 235.701 | *SNRNP27, MXD1, ASPRV1, PCBP1, LOC100133985* | 99 |  |
| **2** | 80,233,408 | 80,307,154 | 73.746 | *CTNNA2* | 101 | Reported |
| **2** | 82,055,473 | 82,163,764 | 108.291 |  | 85 | Reported |
| **2** | 82,056,404 | 82,168,370 | 111.966 |  | 89 | Reported |
| **2** | 95,817,858 | 96,063,153 | 245.295 | *LOC729234, GPAT2* | 68 | Reported |
| **2** | 108,563,678 | 108,624,205 | 60.527 | *LIMS1* | 59 | Reported |
| **2** | 108,597,884 | 108,633,270 | 35.386 | *LIMS1* | 35 |  |
| **2** | 110,707,733 | 111,103,752 | 396.019 | *RGPD6, RGPD5, RGPD7* | 71 | Reported |
| **2** | 117,504,033 | 117,648,418 | 144.385 |  | 55 | Reported |
| **2** | 132,952,949 | 132,972,060 | 19.111 | *GPR39* | 29 |  |
| **2** | 157,969,311 | 158,052,465 | 83.154 | *CYTIP* | 93 |  |
| **2** | 165,336,853 | 165,448,681 | 111.828 | *COBLL1* | 105 | Reported |
| **2** | 194,626,162 | 194,695,204 | 69.042 |  | 40 | Reported |
| **2** | 209,793,755 | 209,821,161 | 27.406 |  | 30 |  |
| **3** | 56,668 | 162,999 | 106.331 |  | 71 | Reported |
| **3** | 243,505 | 293,143 | 49.638 | *CHL1* | 56 | Reported |
| **3** | 958,296 | 1,012,953 | 54.657 |  | 33 | Reported |
| **3** | 975,908 | 1,032,700 | 56.792 |  | 29 | Reported |
| **3** | 1,990,257 | 2,146,421 | 156.164 | *CNTN4* | 155 | Reported |
| **3** | 2,004,482 | 2,185,670 | 181.188 | *CNTN4* | 206 | Reported |
| **3** | 19,661,862 | 19,782,599 | 120.737 |  | 72 | Reported |
| **3** | 21,228,980 | 21,316,691 | 87.711 |  | 90 | Reported |
| **3** | 47,700,789 | 47,835,589 | 134.8 | *SMARCC1, DHX30* | 88 | Reported |
| **3** | 53,121,365 | 53,153,118 | 31.753 | *RFT1* | 35 | Reported |
| **3** | 60,494,885 | 60,632,282 | 137.397 | *FHIT* | 158 |  |
| **3** | 62,936,457 | 62,969,043 | 32.586 |  | 31 | Reported |
| **3** | 101,822,318 | 101,928,257 | 105.939 | *GPR128, TFG* | 97 | Reported |
| **3** | 128,053,684 | 128,084,359 | 30.675 | *CHCHD6* | 25 | Reported |
| **3** | 164,467,838 | 164,648,551 | 180.713 |  | 134 | Reported |
| **3** | 164,844,550 | 164,873,608 | 29.058 |  | 24 | Reported |
| **3** | 166,523,809 | 166,565,186 | 41.377 |  | 39 | Reported |
| **3** | 166,523,809 | 166,565,186 | 41.377 |  | 39 | Reported |
| **3** | 166,523,809 | 166,566,558 | 42.749 |  | 40 | Reported |
| **3** | 166,525,250 | 166,565,186 | 39.936 |  | 38 | Reported |
| **3** | 177,370,126 | 177,396,832 | 26.706 |  | 26 | Reported |
| **4** | 5,704,604 | 5,827,497 | 122.893 | *EVC2, EVC* | 109 | Reported |
| **4** | 21,187,036 | 21,251,815 | 64.779 | *KCNIP4* | 84 | Reported |
| **4** | 21,656,799 | 21,680,565 | 23.766 |  | 28 | Reported |
| **4** | 25,672,202 | 25,703,024 | 30.822 |  | 31 | Reported |
| **4** | 25,678,621 | 25,710,178 | 31.557 |  | 32 | Reported |
| **4** | 25,680,434 | 25,710,412 | 29.978 |  | 31 | Reported |
| **4** | 39,864,888 | 39,888,181 | 23.293 | *RHOH* | 27 |  |
| **4** | 44,661,354 | 44,699,744 | 38.39 |  | 45 | Reported |
| **4** | 44,848,205 | 44,891,375 | 43.17 |  | 31 |  |
| **4** | 57,754,560 | 57,794,496 | 39.936 |  | 28 | Reported |
| **4** | 74,788,136 | 74,829,138 | 41.002 | *IL8* | 47 |  |
| **4** | 74,788,969 | 74,846,104 | 57.135 | *IL8* | 68 |  |
| **4** | 124,157,939 | 124,222,914 | 64.975 | *SPATA5* | 64 |  |
| **4** | 127,645,216 | 127,694,270 | 49.054 |  | 39 |  |
| **4** | 127,816,769 | 127,888,278 | 71.509 |  | 67 | Reported |
| **4** | 135,140,652 | 135,406,723 | 266.071 | *PABPC4L* | 181 | Reported |
| **4** | 160,917,340 | 161,068,954 | 151.614 |  | 119 |  |
| **4** | 160,983,513 | 161,011,918 | 28.405 |  | 29 |  |
| **5** | 12,729,293 | 12,796,179 | 66.886 |  | 59 | Reported |
| **5** | 18,862,917 | 18,986,349 | 123.432 |  | 101 | Reported |
| **5** | 20,765,538 | 21,491,817 | 726.279 |  | 305 | Reported |
| **5** | 53,231,517 | 53,299,530 | 68.013 | *ARL15* | 60 |  |
| **5** | 59,749,693 | 59,807,906 | 58.213 |  | 51 | Reported |
| **5** | 59,749,693 | 59,807,906 | 58.213 |  | 51 | Reported |
| **5** | 59,749,693 | 59,810,944 | 61.251 |  | 52 | Reported |
| **5** | 61,671,081 | 62,120,027 | 448.946 | *KIF2A, DIMT1L, IPO11, LRRC70, ISCA1L* | 348 | Reported |
| **5** | 90,467,891 | 90,519,887 | 51.996 | *GPR98* | 51 |  |
| **5** | 90,473,281 | 90,507,729 | 34.448 | *GPR98* | 39 |  |
| **5** | 109,245,566 | 109,310,548 | 64.982 |  | 44 |  |
| **5** | 110,444,946 | 110,474,788 | 29.842 | *WDR36* | 31 | Reported |
| **5** | 117,705,815 | 117,776,599 | 70.784 |  | 42 | Reported |
| **5** | 119,424,098 | 119,490,200 | 66.102 |  | 81 |  |
| **5** | 123,875,405 | 123,922,152 | 46.747 |  | 25 | Reported |
| **5** | 142,147,309 | 142,174,652 | 27.343 | *ARHGAP26* | 37 |  |
| **5** | 159,756,635 | 159,787,114 | 30.479 | *C5orf54, SLU7, PTTG1* | 33 |  |
| **6** | 11,428,833 | 11,512,036 | 83.203 | *NEDD9* | 48 |  |
| **6** | 20,319,344 | 20,369,570 | 50.226 | *MBOAT1* | 26 |  |
| **6** | 23,008,072 | 23,150,075 | 142.003 |  | 134 | Reported |
| **6** | 26,245,551 | 26,390,396 | 144.845 | *HIST1H1E, HIST1H2BD, HIST1H2BE, HIST1H4D, HIST1H3D, HIST1H2AD, HIST1H2BF, HIST1H4E, HIST1H2BG, HIST1H2AE, HIST1H3E, HIST1H1D, HIST1H4F, HIST1H4G, HIST1H3F, HIST1H2BH, HIST1H3G, HIST1H2BI* | 94 | Reported |
| **6** | 27,738,385 | 27,764,062 | 25.677 |  | 26 |  |
| **6** | 27,742,403 | 27,770,374 | 27.971 |  | 24 |  |
| **6** | 45,108,003 | 45,148,331 | 40.328 | *SUPT3H* | 54 | Reported |
| **6** | 54,210,145 | 54,238,468 | 28.323 | *C6orf142* | 31 |  |
| **6** | 57,021,569 | 57,059,741 | 38.172 | *KIAA1586* | 27 |  |
| **6** | 57,642,203 | 58,179,840 | 537.637 |  | 292 | Reported |
| **6** | 66,447,405 | 66,565,153 | 117.748 | *EYS, MCART3P* | 71 | Reported |
| **6** | 95,438,059 | 95,522,977 | 84.918 |  | 57 | Reported |
| **6** | 108,293,699 | 108,309,380 | 15.681 | *SEC63* | 24 |  |
| **6** | 120,573,785 | 120,843,181 | 269.396 |  | 202 | Reported |
| **6** | 134,579,945 | 134,656,778 | 76.833 | *SGK1* | 48 | Reported |
| **6** | 159,321,956 | 159,460,529 | 138.573 | *RSPH3, TAGAP* | 73 | Reported |
| **6** | 159,373,324 | 159,462,489 | 89.165 | *TAGAP* | 60 |  |
| **7** | 2,946,394 | 2,996,375 | 49.981 | *CARD11* | 41 |  |
| **7** | 7,670,435 | 7,697,631 | 27.196 | *RPA3* | 30 | Reported |
| **7** | 8,635,000 | 8,669,252 | 34.252 | *NXPH1* | 41 |  |
| **7** | 14,070,901 | 14,156,370 | 85.469 | *DGKB* | 49 | Reported |
| **7** | 32,175,629 | 32,389,564 | 213.935 |  | 173 | Reported |
| **7** | 37,684,307 | 37,707,534 | 23.227 |  | 25 | Reported |
| **7** | 54,209,655 | 54,250,179 | 40.524 | *HPVC1* | 35 | Reported |
| **7** | 69,103,254 | 69,121,442 | 18.188 | *AUTS2* | 28 | Reported |
| **7** | 76,267,936 | 76,409,841 | 141.905 |  | 27 | Reported |
| **7** | 76,870,538 | 76,891,903 | 21.365 | *PION* | 27 |  |
| **7** | 78,954,165 | 78,985,428 | 31.263 |  | 32 |  |
| **7** | 79,157,270 | 79,248,166 | 90.896 |  | 64 | Reported |
| **7** | 84,011,749 | 84,159,338 | 147.589 |  | 114 | Reported |
| **7** | 105,672,083 | 105,874,031 | 201.948 | *NAMPT* | 187 | Reported |
| **7** | 105,678,079 | 105,717,255 | 39.176 | *NAMPT* | 43 |  |
| **7** | 105,678,961 | 105,717,255 | 38.294 | *NAMPT* | 42 |  |
| **7** | 105,681,068 | 105,719,044 | 37.976 | *NAMPT* | 42 |  |
| **7** | 105,682,587 | 105,719,044 | 36.457 | *NAMPT* | 40 |  |
| **7** | 110,528,812 | 111,403,681 | 874.869 | *IMMP2L, LRRN3, DOCK4* | 913 | Reported |
| **7** | 110,839,739 | 110,986,789 | 147.05 | *IMMP2L* | 155 | Reported |
| **7** | 112,694,781 | 112,745,252 | 50.471 |  | 45 | Reported |
| **7** | 118,641,078 | 118,818,116 | 177.038 |  | 141 | Reported |
| **7** | 124,931,796 | 125,015,881 | 84.085 |  | 63 | Reported |
| **7** | 125,140,781 | 125,179,835 | 39.054 |  | 46 |  |
| **7** | 130,242,906 | 130,319,788 | 76.882 | *FLJ43663* | 69 |  |
| **8** | 15,929,496 | 15,980,885 | 51.389 |  | 45 | Reported |
| **8** | 15,991,272 | 16,067,860 | 76.588 | *MSR1* | 89 | Reported |
| **8** | 18,752,814 | 18,769,132 | 16.318 | *PSD3* | 26 |  |
| **8** | 27,237,115 | 27,333,842 | 96.727 | *PTK2B* | 101 |  |
| **8** | 42,413,297 | 42,441,326 | 28.029 | *SLC20A2* | 25 |  |
| **8** | 50,594,092 | 50,708,508 | 114.416 |  | 105 | Reported |
| **8** | 59,664,629 | 59,684,769 | 20.14 | *NSMAF* | 26 |  |
| **8** | 91,048,149 | 91,070,004 | 21.855 | *NBN* | 26 | Reported |
| **8** | 91,050,795 | 91,088,236 | 37.441 | *NBN, DECR1* | 25 | Reported |
| **8** | 111,125,017 | 111,200,184 | 75.167 |  | 62 |  |
| **8** | 115,386,302 | 115,443,535 | 57.233 |  | 53 | Reported |
| **8** | 138,007,348 | 138,052,625 | 45.277 |  | 26 | Reported |
| **8** | 139,259,837 | 139,306,535 | 46.698 | *FAM135B* | 40 |  |
| **9** | 12,085,021 | 12,262,520 | 177.499 |  | 82 | Reported |
| **9** | 12,631,342 | 12,809,409 | 178.067 | *TYRP1, C9orf150* | 89 | Reported |
| **9** | 17,572,061 | 17,624,002 | 51.941 | *SH3GL2* | 31 | Reported |
| **9** | 25,249,407 | 25,339,934 | 90.527 |  | 52 | Reported |
| **9** | 30,711,558 | 30,787,411 | 75.853 |  | 44 | Reported |
| **9** | 31,331,953 | 31,398,637 | 66.684 |  | 42 |  |
| **9** | 99,709,291 | 99,742,857 | 33.566 | *C9orf156, HEMGN* | 29 |  |
| **10** | 7,902,891 | 7,984,771 | 81.88 | *TAF3* | 128 |  |
| **10** | 27,644,354 | 27,746,618 | 102.264 | *PTCHD3* | 79 | Reported |
| **10** | 53,053,500 | 53,075,404 | 21.904 | *PRKG1* | 31 |  |
| **10** | 54,943,381 | 55,011,343 | 67.962 |  | 84 | Reported |
| **10** | 56,281,914 | 56,365,117 | 83.203 |  | 67 | Reported |
| **10** | 67,744,533 | 67,785,552 | 41.019 | *CTNNA3* | 48 | Reported |
| **10** | 70,491,424 | 70,537,632 | 46.208 | *SRGN* | 37 |  |
| **10** | 80,654,514 | 80,693,519 | 39.005 | *ZMIZ1* | 28 | Reported |
| **10** | 85,141,591 | 85,204,704 | 63.113 |  | 71 | Reported |
| **10** | 88,875,783 | 89,010,142 | 134.359 | *FAM35A, FAM22A, LOC728190* | 42 | Reported |
| **10** | 125,497,942 | 125,537,290 | 39.348 | *CPXM2* | 48 |  |
| **11** | 31,224,686 | 31,331,311 | 106.625 | *DCDC1* | 62 | Reported |
| **11** | 36,013,603 | 36,054,813 | 41.21 | *LDLRAD3* | 39 | Reported |
| **11** | 37,731,298 | 37,797,204 | 65.906 |  | 49 | Reported |
| **11** | 40,524,347 | 40,601,915 | 77.568 |  | 51 | Reported |
| **11** | 64,948,250 | 65,054,483 | 106.233 | *NCRNA00084, MALAT1, SCYL1* | 25 | Reported |
| **11** | 84,215,785 | 84,247,783 | 31.998 | *DLG2* | 25 | Reported |
| **11** | 85,107,193 | 85,147,570 | 40.377 | *SYTL2* | 51 | Reported |
| **11** | 90,466,127 | 90,514,589 | 48.462 |  | 51 | Reported |
| **11** | 93,922,391 | 93,960,356 | 37.965 | *FUT4, PIWIL4* | 42 |  |
| **11** | 95,844,428 | 95,917,476 | 73.048 |  | 54 | Reported |
| **11** | 95,844,428 | 95,917,476 | 73.048 |  | 54 | Reported |
| **11** | 99,024,813 | 99,074,497 | 49.684 | *CNTN5* | 79 | Reported |
| **11** | 119,679,290 | 119,726,037 | 46.747 | *POU2F3, TMEM136, ARHGEF12* | 52 |  |
| **11** | 119,691,589 | 119,728,609 | 37.02 | *POU2F3, TMEM136, ARHGEF12* | 43 |  |
| **11** | 119,695,754 | 119,724,126 | 28.372 | *POU2F3, TMEM136, ARHGEF12* | 36 |  |
| **11** | 119,697,081 | 119,723,342 | 26.261 | *TMEM136, ARHGEF12* | 33 |  |
| **12** | 8,092,276 | 8,128,782 | 36.506 | *FOXJ2, C3AR1, NECAP1* | 32 | Reported |
| **12** | 14,981,480 | 15,009,411 | 27.931 | *ERP27, ARHGDIB* | 33 |  |
| **12** | 19,364,873 | 19,476,055 | 111.182 | *PLEKHA5* | 37 | Reported |
| **12** | 46,230,956 | 46,333,886 | 102.93 |  | 72 | Reported |
| **12** | 72,795,943 | 72,852,882 | 56.939 |  | 45 |  |
| **12** | 84,574,761 | 85,321,863 | 747.102 | *RASSF9, NTS, MGAT4C* | 577 | Reported |
| **12** | 107,986,861 | 108,071,877 | 85.016 | *USP30, ALKBH2, UNG, ACACB* | 73 | Reported |
| **12** | 108,032,774 | 108,154,981 | 122.207 | *UNG, ACACB* | 76 | Reported |
| **13** | 19,043,727 | 19,211,210 | 167.483 | *MPHOSPH8, PSPC1* | 135 | Reported |
| **13** | 22,437,418 | 23,772,473 | 1335.055 | *SGCG, SACS, TNFRSF19, MIPEP, PCOTH, C1QTNF9B, SPATA13* | 1076 | Reported |
| **13** | 30,177,258 | 30,229,399 | 52.141 | *ALOX5AP* | 53 |  |
| **13** | 45,631,323 | 45,653,717 | 22.394 | *LCP1* | 50 | Reported |
| **13** | 51,225,065 | 51,284,405 | 59.34 | *WDFY2, DHRS12* | 32 | Reported |
| **13** | 56,765,642 | 56,789,452 | 23.81 |  | 25 | Reported |
| **13** | 63,223,695 | 63,320,373 | 96.678 |  | 27 | Reported |
| **13** | 65,996,654 | 66,066,480 | 69.826 | *PCDH9* | 49 | Reported |
| **13** | 66,195,937 | 66,306,972 | 111.035 | *PCDH9* | 95 | Reported |
| **13** | 79,490,715 | 79,565,980 | 75.265 |  | 82 |  |
| **14** | 33,359,188 | 33,601,677 | 242.489 | *EGLN3* | 160 | Reported |
| **14** | 40,520,917 | 40,573,152 | 52.235 |  | 47 | Reported |
| **14** | 44,229,915 | 44,294,996 | 65.081 |  | 53 | Reported |
| **14** | 44,229,915 | 44,294,996 | 65.081 |  | 53 | Reported |
| **14** | 50,387,263 | 50,466,840 | 79.577 | *ABHD12B, PYGL* | 65 |  |
| **14** | 51,375,466 | 51,444,929 | 69.463 | *GNG2* | 77 |  |
| **14** | 51,377,504 | 51,445,958 | 68.454 | *GNG2* | 76 |  |
| **14** | 60,997,821 | 61,156,925 | 159.104 | *PRKCH* | 159 | Reported |
| **14** | 80,659,512 | 80,669,166 | 9.654 | *TSHR* | 32 |  |
| **15** | 42,759,629 | 42,818,430 | 58.801 | *B2M, TRIM69* | 35 | Reported |
| **15** | 42,776,534 | 42,832,003 | 55.469 | *B2M, TRIM69* | 40 | Reported |
| **15** | 49,507,272 | 49,579,058 | 71.786 | *DMXL2* | 70 | Reported |
| **15** | 61,564,457 | 61,599,199 | 34.742 | *USP3* | 32 |  |
| **15** | 79,783,294 | 79,876,946 | 93.652 |  | 77 | Reported |
| **15** | 79,795,446 | 79,876,343 | 80.897 |  | 70 | Reported |
| **15** | 83,259,648 | 83,301,054 | 41.406 | *SLC28A1* | 27 | Reported |
| **15** | 83,622,346 | 83,944,473 | 322.127 | *AKAP13* | 365 | Reported |
| **15** | 87,927,045 | 87,957,524 | 30.479 | *C15orf42* | 33 | Reported |
| **16** | 6,608,417 | 6,629,333 | 20.916 | *A2BP1* | 29 | Reported |
| **16** | 21,449,431 | 21,743,628 | 294.197 | *METTL9, IGSF6, OTOA, LOC730092* | 111 | Reported |
| **16** | 46,156,848 | 46,202,713 | 45.865 | *PHKB* | 56 | Reported |
| **16** | 58,895,868 | 58,912,333 | 16.465 |  | 25 |  |
| **16** | 72,028,260 | 72,119,450 | 91.19 |  | 103 |  |
| **16** | 74,487,570 | 75,293,719 | 806.149 | *CNTNAP4* | 482 | Reported |
| **16** | 76,684,338 | 76,929,109 | 244.771 | *WWOX* | 222 | Reported |
| **16** | 76,947,909 | 77,009,160 | 61.251 | *WWOX* | 69 | Reported |
| **17** | 8,736,773 | 8,820,505 | 83.732 | *PIK3R5* | 68 |  |
| **17** | 8,738,684 | 8,800,866 | 62.182 | *PIK3R5* | 52 |  |
| **17** | 8,756,373 | 8,786,411 | 30.038 | *PIK3R5* | 26 |  |
| **17** | 10,175,413 | 10,239,359 | 63.946 | *MYH13, MYH8* | 90 |  |
| **17** | 19,439,549 | 19,476,055 | 36.506 |  | 28 | Reported |
| **17** | 19,439,549 | 19,476,055 | 36.506 |  | 28 | Reported |
| **17** | 21,503,478 | 21,648,413 | 144.935 |  | 25 | Reported |
| **17** | 21,503,478 | 21,650,626 | 147.148 |  | 26 | Reported |
| **17** | 25,700,671 | 25,756,973 | 56.302 | *CPD* | 52 |  |
| **17** | 26,658,866 | 26,681,211 | 22.345 | *NF1, EVI2B, EVI2A* | 46 | Reported |
| **17** | 27,008,187 | 27,069,683 | 61.496 |  | 57 |  |
| **17** | 41,513,755 | 41,615,467 | 101.712 | *KIAA1267* | 25 | Reported |
| **17** | 42,827,641 | 42,851,015 | 23.374 | *C17orf57* | 33 | Reported |
| **17** | 67,097,537 | 67,128,947 | 31.41 |  | 30 |  |
| **18** | 1,714,779 | 1,828,901 | 114.122 |  | 109 | Reported |
| **18** | 1,714,779 | 1,828,901 | 114.122 |  | 109 | Reported |
| **18** | 1,894,368 | 1,974,284 | 79.916 |  | 63 | Reported |
| **18** | 1,894,368 | 1,974,284 | 79.916 |  | 63 | Reported |
| **18** | 1,894,368 | 1,974,284 | 79.916 |  | 63 | Reported |
| **18** | 1,894,368 | 1,974,284 | 79.916 |  | 63 | Reported |
| **18** | 27,901,310 | 27,931,250 | 29.94 | *RNF125, RNF138* | 29 | Reported |
| **18** | 44,835,024 | 44,858,937 | 23.913 | *DYM* | 31 | Reported |
| **18** | 54,341,024 | 54,380,470 | 39.446 | *ALPK2* | 26 |  |
| **18** | 62,685,430 | 62,726,591 | 41.161 |  | 30 | Reported |
| **18** | 63,997,797 | 64,044,740 | 46.943 |  | 51 | Reported |
| **19** | 36,911,234 | 36,939,557 | 28.323 |  | 36 |  |
| **19** | 36,918,927 | 36,940,929 | 22.002 |  | 32 |  |
| **19** | 36,918,927 | 36,944,555 | 25.628 |  | 36 |  |
| **19** | 47,909,529 | 48,461,673 | 552.144 | *PSG3, PSG8, PSG10, PSG1, PSG6, PSG7, PSG11, PSG2, PSG5, PSG4, PSG9* | 274 | Reported |
| **19** | 54,361,996 | 54,395,072 | 33.076 | *TRPM4* | 26 | Reported |
| **19** | 56,941,209 | 56,979,381 | 38.172 | *FPR1, FPR2* | 63 | Reported |
| **20** | 2,831,979 | 2,944,423 | 112.444 | *PTPRA* | 139 |  |
| **20** | 43,783,582 | 43,811,905 | 28.323 | *SPINT4* | 32 | Reported |
| **20** | 43,783,582 | 43,811,905 | 28.323 | *SPINT4* | 32 | Reported |
| **20** | 51,625,983 | 51,672,877 | 46.894 | *ZNF217* | 29 | Reported |
| **20** | 56,661,174 | 56,681,314 | 20.14 | *STX16* | 24 |  |
| **21** | 15,881,071 | 16,070,408 | 189.337 | *USP25* | 187 | Reported |
| **21** | 18,074,850 | 18,090,874 | 16.024 | *C21orf91* | 24 |  |
| **21** | 28,117,155 | 28,283,805 | 166.65 |  | 55 |  |
| **21** | 29,468,330 | 29,497,829 | 29.499 | *C21orf7, C21orf109* | 28 |  |
| **21** | 38,558,908 | 38,600,216 | 41.308 | *KCNJ15* | 58 | Reported |
| **21** | 40,624,405 | 40,653,316 | 28.911 | *DSCAM* | 37 |  |
| **21** | 40,631,461 | 40,655,668 | 24.207 | *DSCAM* | 32 |  |
| **22** | 24,064,463 | 24,182,260 | 117.797 | *LRP5L* | 24 | Reported |
| **22** | 24,251,398 | 24,330,534 | 79.136 | *ADRBK2* | 87 | Reported |
| **22** | 33,098,479 | 33,209,980 | 111.501 |  | 159 | Reported |

**Table S8: List of 67 genes associated with CNVs uniquely identified in patients and not yet associated with malignancy .**

| **Novel genes associated with BC** | | | | | |
| --- | --- | --- | --- | --- | --- |
| *ABHD12B* | *C6orf142* | *GPR98* | *NEDD9* | *SEC63* | *USP3* |
| *ACYP2* | *C9orf156* | *HEMGN* | *NSMAF* | *SLC20A2* | *ZNF436* |
| *ALOX5AP* | *CAMK1G* | *HSD11B1* | *NXPH1* | *SLU7* |  |
| *ALPK2* | *CDC42SE1* | *IL8* | *PCBP1* | *SMAP2* |  |
| *ARHGDIB* | *CPXM2* | *KIAA1586* | *PIK3R5* | *SNRNP27* |  |
| *ARL15* | *CYTIP* | *LAMB3* | *PION* | *SPATA5* |  |
| *ASPRV1* | *DSCAM* | *LIMS1* | *PIWIL4* | *SRGN* |  |
| *C1orf213* | *ERP27* | *LOC100133985* | *POU2F3* | *STX16* |  |
| *C1orf56* | *FLJ43663* | *MBOAT1* | *PRKG1* | *TAF3* |  |
| *C21orf109* | *FUT4* | *MXD1* | *PSD3* | *TAGAP* |  |
| *C21orf7* | *G0S2* | *MYH13* | *PTPRA* | *TCEA3* |  |
| *C21orf91* | *GNG2* | *MYH8* | *PTTG1* | *TMEM136* |  |
| *C5orf54* | *GPR39* | *NAMPT* | *PYGL* | *TSPYL6* |  |

**References**

1. Cancer Gene Census. (Cancer Genome Project Wellcome Trust Sanger Institute, 2012).

2. D'Antonio, M., Pendino, V., Sinha, S. & Ciccarelli, F.D. Network of Cancer Genes (NCG 3.0): integration and analysis of genetic and network properties of cancer genes. *Nucleic Acids Res* **40**, D978-83 (2012).
